# Features of Helium–Vacancy Complex Formation at the Zr/Nb Interface

**DOI:** 10.3390/ma16103742

**Published:** 2023-05-15

**Authors:** Leonid Svyatkin, Daria Terenteva, Roman Laptev

**Affiliations:** Division for Experimental Physics, National Research Tomsk Polytechnic University, 634050 Tomsk, Russia; svyatkin@tpu.ru (L.S.); dvt17@tpu.ru (D.T.)

**Keywords:** nanoscale multilayer coatings, helium, vacancy, zirconium/niobium interface, density functional theory

## Abstract

A first-principles study of the atomic structure and electron density distribution at the Zr/Nb interface under the influence of helium impurities and helium–vacancy complexes was performed using the optimised Vanderbilt pseudopotential method. For the determination of the preferred positions of the helium atom, the vacancy and the helium–vacancy complex at the interface, the formation energy of the Zr-Nb-He system has been calculated. The preferred positions of the helium atoms are in the first two atomic layers of Zr at the interface, where helium–vacancy complexes form. This leads to a noticeable increase in the size of the reduced electron density areas induced by vacancies in the first Zr layers at the interface. The formation of the helium–vacancy complex reduces the size of the reduced electron density areas in the third Zr and Nb layers as well as in the Zr and Nb bulk. Vacancies in the first niobium layer near the interface attract the nearest zirconium atoms and partially replenish the electron density. This may indicate a possible self-healing of this type of defect.

## 1. Introduction

The impact of high-energy particles such as protons, neutrons, helium and lithium ions on metals leads to intense structural changes caused by the displacement of atoms with the formation of primary radiation defects. At sufficient concentrations, primary radiation defects can form more complex defects, such as dislocation loops, stacking faults, interstitials and vacancy clusters [1]. In the presence of hard-soluble gases, clusters can develop into stable pores and promote the formation of gas bubbles. All of these defects cause radiation swelling and embrittlement, which significantly degrade the material properties [2]. This problem is particularly acute in the nuclear industry, where structural materials are exposed to high temperatures, high mechanical loads, chemically aggressive coolants and intense radiation fluxes.

Obviously, a reduction of the primary radiation defects will suppress further development of the defect structure. A high level of radiation resistance in materials can be achieved by using free surfaces, grain boundaries and heterophase interfaces as efficient radiation defect sink sites [3,4]. Nanoscale multilayer metallic systems have gained particular interest because of the possibility of varying the metallic components to form different types of interfaces with exceptional physical and mechanical properties. Interface coherence and metal miscibility are the main factors that determine the efficiency and stability of a multilayer system under irradiation.

The effectiveness of the interface as a sink for defects is determined by the interface coherence, which is dependent on the crystal type, structure type and the lattice parameter difference. An incoherent or semi-coherent interface is an efficient sink for defects, due to the presence of a large number of mismatched dislocations. Since intense exposure to high-energy particles can result in metal mixing and interface destruction, the layer’s miscibility mainly determines the morphological stability of the system. Thus, incoherent or semi-coherent systems with low layer miscibility will be the most promising [5,6,7,8,9,10,11,12,13,14]. One of the most suitable systems is a system based on zirconium and niobium, since these materials are widely used in the nuclear industry due to good mechanical and corrosion properties, as well as low thermal neutron capture cross-section [15]. Zirconium and niobium can form various types of interfaces due to the different crystal lattices (hcp and bcc) and positive mixing enthalpy (4 kJ/mol) [16]. The high radiation tolerance of nanoscale multilayer Zr/Nb systems has been demonstrated in recent studies, including helium ion irradiation [17,18,19,20,21,22,23,24,25]. Helium atoms are produced in materials as a result of nuclear reactions (n, α) [26] and tend to accumulate in vacancies and interstitials with the formation of helium bubbles, leading to changes in the macroscopic properties of the irradiated material [27,28,29,30,31,32,33]. However, the accumulation of helium atoms at the Zr/Nb interface and their effect on the atomic and electronic structure of metals remain incompletely understood. Moreover, since vacancies are effective trapping centres for helium atoms, it is also necessary to consider the formation of helium–vacancy complexes at the Zr/Nb interface.

The purpose of this work is to study by ab initio methods the atomic structure and electron density distribution of metals at the Zr/Nb interface when a helium impurity is introduced and a helium–vacancy complex is formed.

## 2. Methods and Details of Calculation

Ab initio calculations were carried out within density functional theory using the optimised norm-conserving Vanderbilt pseudopotential method [34], as implemented in the ABINIT code [35,36]. The generalised gradient approximation (GGA) in the form proposed by Perdew, Burke and Ernzerhof [37] was used to describe the exchange and correlation effects. In the structural optimisation, the cutoff energy for the plane wave basis was set to 15 Ha. The atoms in the system were assumed to be in the equilibrium configuration when the force on each atom was less than 10^−3^ Ha/Bohr.

**Figure 1:**
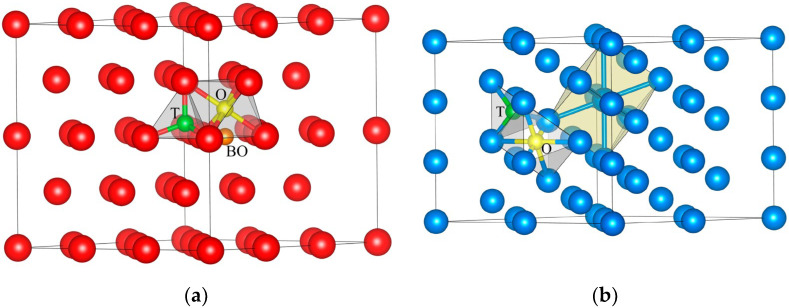
The interstitial sites in the (**a**) zirconium and (**b**) niobium supercells. Tetrahedral sites are green, octahedral sites are yellow. In hcp zirconium BO site is orange.

In our previous work [38] the helium atom in the zirconium lattice was located in the tetrahedral (T) and octahedral (O) sites, in the interstitial site of the basal plane (BO) and in the vacancy (vac) with the defect concentration of 3 at.% (Figure 1a).

The interstitial site BO and vacancy positions of the He atom were found to be the most stable configurations with the lowest formation energy. Due to its metastability in the Zr lattice, the O site was not further taken into consideration. In this work the pure Nb and Nb_36_He solid solutions with helium in tetrahedral or octahedral interstitial sites were considered (Figure 1b). To carry out the structural optimisation and relaxation of the considered system, a cell with 36 Nb atoms was adopted, and the *k* meshes were chosen to be 3 × 3 × 3 for the bcc structure.

**Figure 2:**
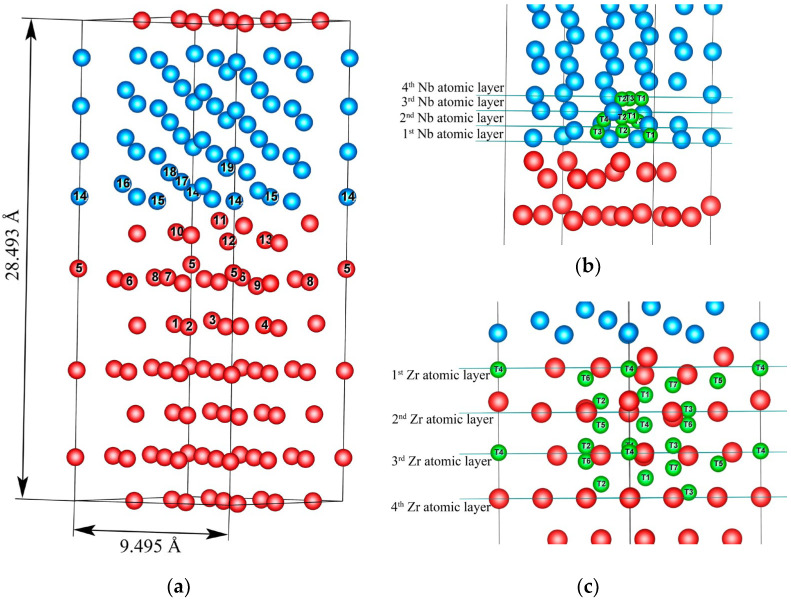
The supercell of the Zr_63_Nb_40_ multilayer structures (**a**) and positions of the interstitial sites considered in the Zr_63_Nb_40_ supercell: (**b**) in Nb layer, (**c**) in Zr layer. Zirconium atoms are red, niobium atoms are blue, tetrahedral sites are green.

The present calculations were performed for Zr_63_Nb_40_ and Zr_63_Nb_40_He slabs (Figure 2). The interface in the Zr_63_Nb_40_ slab was formed by Zr (002) and Nb (111) surfaces. There are 7 and 10 atomic layers, respectively, in the Zr and Nb slabs. In our earlier work [23], we described the features of the slab supercell structure and the relaxation of its atoms. In the Zr_63_Nb_40_He system a He atom is located only in the tetrahedral (T) interstitial sites. For a more convenient discussion of the results, the T sites are enumerated in Figure 2b,c. For Zr_63_Nb_40_ multilayer structures, *k* meshes of 3 × 3 × 1 were chosen.

To estimate the structural stability of the above-mentioned systems, the formation energy of the system with a helium atom, vacancy and helium–vacancy complex was calculated:

*E*_f_ = *E*(Zr_m_Nb_n_He) − *E*(He) − *E*(Zr_m_Nb_n_), (1)

*E*_vac_ = *E*(Zr_m−y_Nb_n−x_) + *y*×*E*(Zr_m_)/*m* + *x*×*E*(Nb_n_)/*n* − *E*(Zr_m_Nb_n_), (2)

*E*_He-vac_ = *E*(Zr_m−y_Nb_n−x_He) + *y*×*E*(Zr_m_)/*m* + *x*×*E*(Nb_n_)/*n* − *E*(He) − *E*(Zr_m_Nb_n_). (3)

Here, *E*(Zr_m−y_Nb_n−x_) and *E*(Zr_m_Nb_n_) are the total energies of the systems consisting of *m* Zr and *n* Nb atoms with and without vacancies, respectively, *x* and *y* indicate the number of vacancies in Nb and Zr, respectively, *E*(Zr_m−y_Nb_n−x_He) and *E*(Zr_m_Nb_n_He) are the total energies of systems consisting of a He atom, *m* Zr and *n* Nb atoms with and without vacancies, respectively, *E*(He) is the total energy of an isolated helium atom.

## 3. Results and Discussion

The formation energies of Zr_36_He and Nb_36_He solid solutions with interstitial helium are equal to 2.699 and 2.433, 3.170 and 3.438 eV for the He atom in the T and BO sites of the hcp Zr lattice and the T and O sites of the bcc Nb lattice, respectively. These results are in good agreement with the results of other calculations [39,40,41]. Due to the large Zr lattice distortions near the interface [23] and the significant difference between the Nb_36_He^T^ and Nb_36_He^O^ formation energies (0.268 eV) the only tetrahedral coordination of the He atom in the Zr_63_Nb_40_He slab was considered (see Figure 2b,c).

The calculated vacancy formation energies for the hcp Zr and bcc Nb lattices are 2.069 и 2.687 eV, which are also in a good agreement with previous work [39,42,43,44,45,46]. In the hcp Zr and bcc Nb lattices, helium–vacancy complex formation requires 3.320 eV and 4.329 eV, respectively. It should be noted that the difference between the vacancy formation energy and the helium–vacancy complex formation energy gives the energy required to place the helium atom into the pre-existing vacancy.

The calculated formation energies of the Zr_63_Nb_40_He system with interstitial helium are given in Table 1. The significant variation in formation energy values is observed for the position of the He atom in both the Zr and Nb atomic layers. For instance, the formation energy in the first zirconium layer varies from 1.741 eV to 2.760 eV. For niobium the variation of the formation energy value seems to be less, for example, for the first niobium atomic layer the minimum of the formation energy is 1.754 eV and the maximum is 2.452 eV. The detailed analysis of the electron density distribution in the Zr_63_Nb_40_ slab showed that in the most energetically favourable position (position (4), (5) and (1)) in the first Zr atomic layer the helium atom shifts due to relaxation to the area with the low value of the electron density. This behaviour is explained by the fact that filled He 1*s* electron states displace metal electrons from the region where the He atom is located, leading to a noticeable redistribution of the electron density of the system. The same observations are applied to the first Nb layer: the relaxation of the helium atom in the (1) site leads to its shift to the nearest reduced electron density area. Due to the relaxation in the (1) position, the helium atom was shifted from the niobium layer to the zirconium one, occupying the (4) position in the first Zr layer. This explains the similar values of the formation energies of the Zr_63_Nb_40_He system with the He atom in (4) site in the first Zr layer and (1) site in the first Nb layer. The helium from (3) site in the second Nb layer also shifted into the interface due to relaxation. It can be concluded that the helium occupation of zirconium sites is more favourable than that of niobium sites. In addition, it should be noted that the helium atom from (4) positions in the second and the third Zr layers is shifted to a layer closer to the interface due to relaxation.

**Figure 3:**
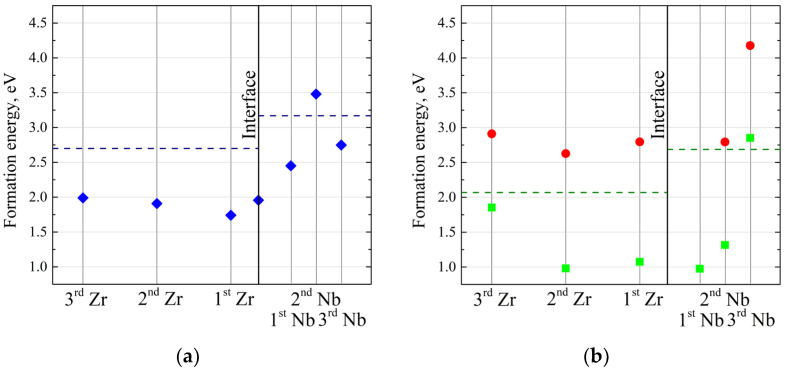
The dependence of the minimal values of the formation energy of the Zr_63_Nb_40_He system with interstitial helium (**a**), the vacancy formation energy (green squares) and the helium–vacancy complex formation energy (red dots) (**b**) on the atomic layer number. The dark blue dotted line indicates the value of the formation energy in T sites and the dark green dotted line indicates the value of the vacancy formation energy for both pure Zr and Nb.

Figure 3 displays the lowest value of formation energy for both metals, layer by layer near the interface. This allows us to consider the most energetically favourable interstitial positions for the helium atom. It can be seen that the minimum layer-by-layer formation energy increases significantly in niobium compared to zirconium. It can be expected that for the layers far from the interface this formation energy should diverge towards the formation energy value in the solid material. This is more obvious for the niobium: in the third Nb layer the minimal formation energy is 2.750 eV while the same formation energy in the Nb bulk is 3.170 eV. For zirconium, however, this assumption is not appropriate due to the large intralayer variation which depends on the atomic relaxation and chemical bonds. However, considering the formation energies in the third atomic layer of Zr, it can be seen that most of these formation energy values (see Table 1) are comparable to the value for the bulk.

The calculated vacancy formation energies for the Zr_63_Nb_40_ slab are shown in Table 2. The vacancy formation energy is affected by the size of the formed region with a reduced electron density. Thus, the larger the value of the vacancy formation energy, the larger the size of the formed void with a lack of electrons. From the table it can be seen that the variation of the vacancy formation energy is insignificant. Therefore, the void volumes are almost the same. It should be noted that in general no vacancy shift is observed for (1), (2) and (3) vacancy positions. However, in the case of (4) vacancy position, atoms from the upper layers are directed towards this vacancy to fill it. The variation of the formation energy from 0.980 eV to 2.586 eV in the second Zr layer is observed. During relaxation, the vacancy in (8) position moved from the second Zr layer to the first Zr layer. In the remaining cases the vacancies remain static, i.e., they remain in the positions where they were formed. In the first Zr layer for the most energetically favourable (13) vacancy position the nearest neighbours from the second Zr layer and the first Nb layer rushed towards the vacancy. For the remaining cases in this layer no relaxation features are observed. Finally, the atom from the first layer tends to fill a vacancy in the second layer. When a vacancy is formed in the third layer, the atoms from the overlying layers are displaced towards the vacancy.

As for the vacancy positions in the first and second Nb layers, the reduced electron density area formed by the vacancy combines with the reduced electron density area in the first zirconium layers or interface region. Vacancy formation in the third Nb layer leads to large void formation. However, it should be noted that for the most energetically favourable (15) vacancy position in the first Nb layer the nearest Zr atoms from the first layer are shifted (by 1.788 Å) towards the formed vacancy. Thus, the significant influence of the interface on the vacancy formation energy is limited to the first two layers of zirconium and niobium. In the third zirconium or niobium layer from the interface the vacancy formation energies are already comparable to the bulk values. This means that it is energetically more advantageous for the vacancies to be in the vicinity of the interface, where their effect on the atomic structure is suppressed by the interface. These results are indirectly confirmed by experimental studies of the microstructure of Zr/Nb nanoscale multilayer coatings irradiated with helium ions [25], which revealed different microstrains and distortions in the Zr and Nb layers and a different localisation of the implanted ions within the interfaces without redundant radiation defect accumulation.

After considering the vacancy position, the helium atom was placed in the positions instead of Zr and Nb atoms where the vacancy formation energy is the lowest. The results of the helium–vacancy complex formation energy for the most energetically favourable vacancy configurations are presented in Figure 3. From the analysis of the results presented in Figure 3, it follows that the vacancy formation and helium–vacancy complex formation energies increase with distance from the interface in the Zr_63_Nb_40_ slab. However, it should be noted that the growth rate of the formation energies in zirconium is much lower than in niobium. It was established that helium is shifted from the first and second Nb layers to the first layer of zirconium during relaxation. Small variations in the helium–vacancy complex formation energy for the position of the helium atom in the first two layers of zirconium and niobium are observed due to different sizes of voids with a lack of electrons around the helium atom. Since the vacancies in zirconium are located one below the other, the helium atom is shifted from the second zirconium layer to the first zirconium layer. We can assume that the vacancy occupation by helium atoms in zirconium is more favourable than that in niobium. It is shown that from the third layer of both zirconium and niobium the helium atom is not shifted.

**Figure 4:**
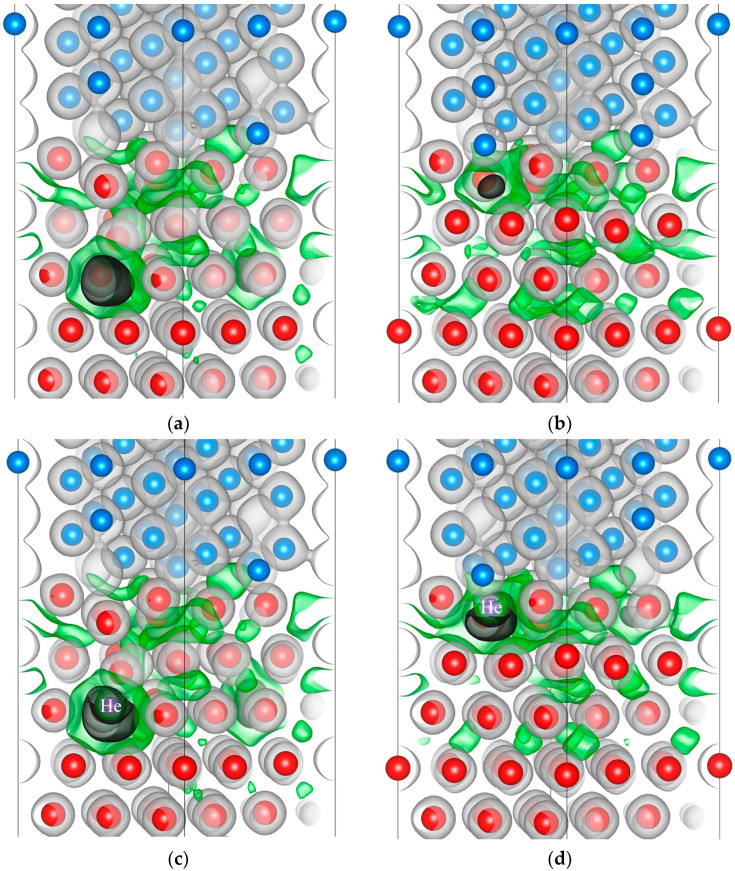
The electron density distribution in the interface vicinity of the Zr_63_Nb_40_ slab with a vacancy (**a**,**b**) and helium–vacancy complex (**c**,**d**) in the third (**a**,**c**) and first Zr layers (**b**,**d**). Zirconium atoms are red, niobium atoms are blue, helium atom is purple. Black, green and grey isosurfaces correspond to the electron density of 0.01, 0.02 and 0.05 electrons/Bohr^3^, respectively.

For qualitative analysis of the influence of a vacancy and a helium–vacancy complex on the interaction between metal atoms at the Zr/Nb interface, the valence electron density distribution in the Zr_63_Nb_40_ slab was calculated. From the analysis of the electron density distribution in the Zr_63_Nb_40_He system with interstitial helium, it was found that the presence of the helium atom in interstitial sites of the metal lattice leads to an insignificant variation of the electron density. Therefore, the consideration of vacancies and helium–vacancy complexes is of greater interest. The electron density distribution in the interface vicinity in the Zr_63_Nb_40_ slab with a vacancy and a helium–vacancy complex in the most energetically favourable position in the third and first Zr layers is shown in Figure 4.

**Figure 5:**
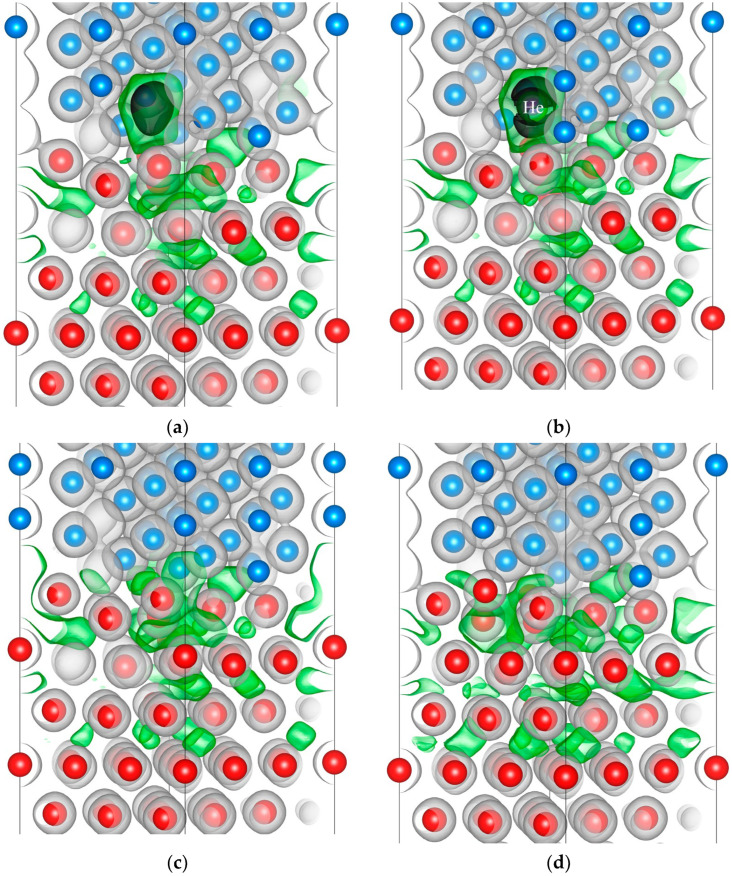
The electron density distribution in the interface vicinity of the Zr_63_Nb_40_ slab with a vacancy (**a**,**c**,**d**) and helium–vacancy complex (**b**) in the third (**a**) and first Nb layers (vacancy is formed in (14) (**c**) and (15) (**d**) positions). Zirconium atoms are red, niobium atoms are blue, helium atom is purple. Black, green and grey isosurfaces correspond to the electron density of 0.01, 0.02 and 0.05 electrons/Bohr^3^, respectively.

The formation of the vacancy considered in both cases leads to the appearance of the reduced electron density area (the electron density in these areas is less than 0.01 electrons/Bohr^3^). The addition of a He atom to the vacancy increases the size of this area in the first Zr layer and decreases it in the third Zr layer (Figure 5). It should be noted that the He atom is located at the periphery of the reduced electron density area formed by vacancies. The same results were observed in Zr-He solid solution with a helium–vacancy complex in our previous work [38]: the helium atom is located in the position shifted from the vacancy along the hexagonal axis. Thus, the helium atom located in the vacancy in the first Zr layer at the interface enhances the electron density redistribution region caused by the vacancy formation. An increase in the size of the reduced electron density area indirectly indicates a weakening of the bonds between the nearest metal atoms, which may contribute to the self-healing properties of the interface due to the higher mobility of weakly bound metal atoms. 

The electron density distribution at the interface of the Zr_63_Nb_40_ slab with a vacancy in the third and first Nb layers and helium–vacancy complex in the third Nb layer is shown in Figure 5. Since it has been previously established that a helium atom does not form the helium–vacancy complex in the first Nb layer at the Zr/Nb interface, we have considered only the electron density distribution caused by a vacancy in the first Nb layer of the Zr_63_Nb_40_ slab.

The analysis of the electron density distribution in Zr_63_Nb_40_ with a vacancy and a helium–vacancy complex in the third Nb layer (Figure 5a,b) shows that the formation of a vacancy and a helium–vacancy complex leads to the same results as described above for the third Zr layer. However, in the Nb layer the He atom is located in the centre of the vacancy. From Figure 5c,d it was established that the vacancy in the first niobium layer near the interface attracts the nearest zirconium atoms partially refilling the electron density. It prevents the formation of helium–vacancy complexes in the first layer of Nb, because it is not energetically favourable for the helium atom to be in the high electron density region: the helium atom has a filled 1*s* shell and its position in the interstitial region of the lattice causes the electron density of the metal to be pushed out of its vicinity, increasing the total energy of the system. This causes the helium atom to move from the vacancy in the first layer of niobium to the zirconium layer.

Thus, the radiation tolerance of the Zr/Nb nanoscale multilayers under helium ion irradiation is provided by two effects: (1) vacancies formed in layers closest to the Zr/Nb interface are subdued by substantial relaxation of metal atoms at the interface; (2) helium atoms form helium–vacancy complexes predominantly in zirconium layers closest to the interface, increasing the mobility of Zr atoms through bond weakening, which promotes enhanced defect annihilation. These effects can lead to self-healing of defects formed due to irradiation of nanoscale multilayered Zr/Nb layers.

## 4. Conclusions

A first-principles study of the atomic structure and electron density distribution of metals at the Zr (200)/Nb (111) interface under the influence of helium impurities and helium–vacancy complexes was performed. The optimized norm-conserving Vanderbilt pseudopotential method was used for all calculations within density functional theory. The formation energy of the Zr-Nb-He system was calculated to determine the energetically favourable position of the helium atom, the vacancy and the helium–vacancy complex at the Zr/Nb interface. The energetically more favourable helium atom positions were found in the first two Zr atomic layers at the interface, where helium–vacancy complexes were formed. It was revealed that a helium atom in the most considered cases is displaced from the first two layers of Nb atoms to the first layer of Zr atoms, hence its position in this Nb atomic layer is unstable. This leads to a noticeable increase in the size of the reduced electron density areas induced by vacancies in the first Zr layers at the interface. In the other Zr layers and in the third layer of Nb, helium–vacancy complex formation decreases the size of the reduced electron density areas as in the Zr and Nb bulk.

The formation of a helium–vacancy complex leads to a considerable increase in the size of the reduced electron density area (density is less than 0.01 electrons/Bohr^3^) induced by vacancies in the first Zr layers at the interface. In the third Zr and Nb layers the helium-vacancy complex formation decreases the size of the reduced electron density areas as in the Zr and Nb bulk. It was shown that the vacancy in the first layer of niobium near the interface attracts the nearest zirconium atoms and partially refills the electron density. It prevents the formation of helium–vacancy complexes in the first Nb layer. Thus, only the first two zirconium and niobium layers are significantly influenced by the interface for the formation of vacancy and the helium–vacancy complex. In the third zirconium or niobium layer the formation energy and electron density distribution are already at a level close to the bulk values.

## Figures and Tables

**Table 1:** The formation energy of the Zr_63_Nb_40_He system with interstitial helium.

No.	E_f_ (eV)					
	Zr			Nb		
	in 1st Atomic Layer	in 2nd Atomic Layer	in 3rd Atomic Layer	in 1st Atomic Layer	in 2nd Atomic Layer	in 3rd Atomic Layer
1	1.911	2.486	2.371	1.754	3.480	3.379
2	2.392	2.331	2.759	2.451	2.457	3.321
3	2.760	2.996	2.820	2.452	1.957	2.749
4	1.741	1.909	1.990	-	2.456	3.395
5	1.764	2.379	2.561	-	-	-
6	2.125	2.530	2.863	-	-	-
7	2.559	-	2.777	-	-	-

**Table 2:** The vacancy formation energy for the Zr_63_Nb_40_ slab.

Vacancy Number	E_vac_ (eV)	Vacancy Number	E_vac_ (eV)	Vacancy Number	E_vac_ (eV)	Vacancy Number	E_vac_ (eV)	Vacancy Number	E_vac_ (eV)	Vacancy Number	E_vac_ (eV)
Vacancy in Zr						Vacancy in Nb					
1st Layer		2nd Layer		3rd Layer		1st Layer		2nd Layer		3rd Layer	
10	2.543	5	2.130	1	1.953	14	0.976	16	2.029	18	3.026
11	1.760	6	0.980	2	1.958	15	0.532	17	1.317	19	2.883
12	1.074	7	2.586	3	1.873	-	-	-	-	-	-
13	1.074	8	0.936	4	1.705	-	-	-	-	-	-
-	-	9	1.713	-	-	-	-	-	-	-	-

## Data Availability

The data presented in this study are available on request from the corresponding author. The data are not publicly available due to privacy reasons.

## References

[1] Ullmaier H. (1997). Radiation Damage in Metallic Materials. MRS Bull..

[2] Was G.S., Petti D., Ukai S., Zinkle S. (2019). Materials for Future Nuclear Energy Systems. J. Nucl. Mater..

[3] Yang W., Pang J., Zheng S., Wang J., Zhang X., Ma X. (2019). Interface Effects on He Ion Irradiation in Nanostructured Materials. Materials.

[4] Khanal L.R., Sundararajan J.A., Qiang Y. (2019). Advanced Nanomaterials for Nuclear Energy and Nanotechnology. Energy Technol..

[5] Xue J., Li Y., Gao L., Qian D., Song Z., Wang X., Zhu X., Chen J. (2020). Effects of Periods on the Evolution of Microstructure and Mechanical Properties of Multilayered Cu-W Films during Thermal Annealing. Surf. Coat. Technol..

[6] Li M., Hou Q., Cui J., Qiu M., Yang A., Zhou M. (2021). Atomistic Simulations of Helium Behavior at the Cu(111)/W(110) Interface. J. Nucl. Mater..

[7] Cui Y., Derby B., Li N., Misra A. (2021). Fracture Resistance of Hierarchical Cu–Mo Nanocomposite Thin Films. Mater. Sci. Eng. A.

[8] Li S.-H., Li J.-T., Han W.-Z. (2019). Radiation-Induced Helium Bubbles in Metals. Materials.

[9] Ni J., Li J., Jian J., He J., Chen H., Leng X., Liu X. (2021). Recent Studies on the Fabrication of Multilayer Films by Magnetron Sputtering and Their Irradiation Behaviors. Coatings.

[10] Su Z., Jiang H., Li H., Zhang Y., Chen J., Zhao J., Ma Y. (2022). Recent Progress on Interfaces in Nanomaterials for Nuclear Radiation Resistance. ChemNanoMat.

[11] Qi N., Zhang H.X., Chen Z.Q., Ren F., Zhao B., Jiang M., Uedono A. (2020). Selective Trapping of Positrons by Ag Nanolayers in a V/Ag Multilayer System. AIP Adv..

[12] Yang Z., Qiu N., Yang H., Chen Q., Wang Y. (2023). Irradiation Tolerance Enhanced by Coherent Interfaces of FCC/BCC HEA Multilayers. Surf. Coat. Technol..

[13] Chen H., Du J., Liang Y., Wang P., Huang J., Zhang J., Zhao Y., Wang X., Zhang X., Wang Y. (2019). Comparison of Vacancy Sink Efficiency of Cu/V and Cu/Nb Interfaces by the Shared Cu Layer. Materials.

[14] Anwar Ali H.P., Radchenko I., Li N., Budiman A. (2019). Effect of Multilayer Interface through in Situ Fracture of Cu/Nb and Al/Nb Metallic Multilayers. J. Mater. Res..

[15] Jiang G., Xu D., Yang W., Liu L., Zhi Y., Yang J. (2022). High-Temperature Corrosion of Zr–Nb Alloy for Nuclear Structural Materials. Prog. Nucl. Energy.

[16] Debski A., Debski R., Gasior W. (2014). New Features of Entall Database: Comparison of Experimental and Model Formation Enthalpies/ Nowe Funkcje Bazy Danych Entall: Porównanie Doświadczalnych I Modelowych Entalpii Tworzenia. Arch. Metall. Mater..

[17] Daghbouj N., Sen H.S., Callisti M., Vronka M., Karlik M., Duchoň J., Čech J., Havránek V., Polcar T. (2022). Revealing Nanoscale Strain Mechanisms in Ion-Irradiated Multilayers. Acta Mater..

[18] Sen H.S., Daghbouj N., Callisti M., Vronka M., Karlík M., Duchoň J., Čech J., Lorinčík J., Havránek V., Bábor P. (2022). Interface-Driven Strain in Heavy Ion-Irradiated Zr/Nb Nanoscale Metallic Multilayers: Validation of Distortion Modeling via Local Strain Mapping. ACS Appl. Mater. Interfaces.

[19] Liang X.Q., Wang Y.Q., Zhao J.T., Wu S.H., Feng X.B., Wu K., Zhang J.Y., Liu G., Sun J. (2019). Size-Dependent Microstructure Evolution and Hardness of He Irradiated Nb/Zr Multilayers under Different Ion Doses. Mater. Sci. Eng. A.

[20] Daghbouj N., Sen H.S., Čížek J., Lorinčík J., Karlík M., Callisti M., Čech J., Havránek V., Li B., Krsjak V. (2022). Characterizing Heavy Ions-Irradiated Zr/Nb: Structure and Mechanical Properties. Mater. Des..

[21] Daghbouj N., Callisti M., Sen H.S., Karlik M., Čech J., Vronka M., Havránek V., Čapek J., Minárik P., Bábor P. (2021). Interphase Boundary Layer-Dominated Strain Mechanisms in Cu+ Implanted Zr-Nb Nanoscale Multilayers. Acta Mater..

[22] Laptev R., Stepanova E., Pushilina N., Svyatkin L., Krotkevich D., Lomygin A., Ognev S., Siemek K., Doroshkevich A., Uglov V. (2022). Distribution of Hydrogen and Defects in the Zr/Nb Nanoscale Multilayer Coatings after Proton Irradiation. Materials.

[23] Laptev R., Svyatkin L., Krotkevich D., Stepanova E., Pushilina N., Lomygin A., Ognev S., Siemek K., Uglov V. (2021). First-Principles Calculations and Experimental Study of H^+^-Irradiated Zr/Nb Nanoscale Multilayer System. Metals.

[24] Laptev R., Lomygin A., Krotkevich D., Syrtanov M., Kashkarov E., Bordulev Y., Siemek K., Kobets A. (2020). Effect of Proton Irradiation on the Defect Evolution of Zr/Nb Nanoscale Multilayers. Metals.

[25] Laptev R., Stepanova E., Pushilina N., Kashkarov E., Krotkevich D., Lomygin A., Sidorin A., Orlov O., Uglov V. (2023). The Microstructure of Zr/Nb Nanoscale Multilayer Coatings Irradiated with Helium Ions. Coatings.

[26] Huang J., Liu H., Gao Z., Su Y., Liu Q., Ge W., Luo F., Xia S., Cao L., Xue J. (2022). Helium-Hydrogen Synergistic Effects in Structural Materials Under Fusion Neutron Irradiation. Front. Mater..

[27] Yang H.L., Kano S., McGrady J., Chen D.Y., Murakami K., Abe H. (2020). Microstructural Evolution and Hardening Effect in Low-Dose Self-Ion Irradiated Zr–Nb Alloys. J. Nucl. Mater..

[28] Yang H. (2022). Anisotropic Effects of Radiation-Induced Hardening in Nuclear Structural Materials: A Review. J. Nucl. Mater..

[29] Maxwell C., Pencer J., Torres E. (2020). Atomistic Simulation Study of Clustering and Evolution of Irradiation-Induced Defects in Zirconium. J. Nucl. Mater..

[30] King D.J.M., Knowles A.J., Bowden D., Wenman M.R., Capp S., Gorley M., Shimwell J., Packer L., Gilbert M.R., Harte A. (2022). High Temperature Zirconium Alloys for Fusion Energy. J. Nucl. Mater..

[31] Li Y., French A., Hu Z., Gabriel A., Hawkins L.R., Garner F.A., Shao L. (2023). A Quantitative Method to Determine the Region Not Influenced by Injected Interstitial and Surface Effects during Void Swelling in Ion-Irradiated Metals. J. Nucl. Mater..

[32] Zhu H., Qin M., Wei T., Davis J., Ionescu M. (2023). Atomic-Scale Study of He Ion Irradiation-Induced Clustering in α-Zirconium. Acta Mater..

[33] Dai C., Varvenne C., Saidi P., Yao Z., Daymond M.R., Béland L.K. (2021). Stability of Vacancy and Interstitial Dislocation Loops in α-Zirconium: Atomistic Calculations and Continuum Modelling. J. Nucl. Mater..

[34] Hamann D.R. (2013). Optimized Norm-Conserving Vanderbilt Pseudopotentials. Phys. Rev. B.

[35] Gonze X., Amadon B., Antonius G., Arnardi F., Baguet L., Beuken J.-M., Bieder J., Bottin F., Bouchet J., Bousquet E. (2020). The Abinit project: Impact, Environment and Recent Developments. Comput. Phys. Commun..

[36] Romero A.H., Allan D.C., Amadon B., Antonius G., Applencourt T., Baguet L., Bieder J., Bottin F., Bouchet J., Bousquet E. (2020). ABINIT: Overview and Focus on Selected Capabilities. J. Chem. Phys..

[37] Perdew J.P., Burke K., Ernzerhof M. (1996). Generalized Gradient Approximation Made Simple. Phys. Rev. Lett..

[38] Svyatkin L.A., Terenteva D.V., Laptev R.S. (2021). Influence of Vacancy on Helium Interaction with α-Zirconium. J. Phys. Conf. Ser..

[39] Sen H.S., Polcar T. (2019). Vacancy-Interface-Helium Interaction in Zr-Nb Multi-Layer System: A First-Principles Study. J. Nucl. Mater..

[40] Kashinath A., Demkowicz M.J. (2011). A Predictive Interatomic Potential for He in Cu and Nb. Model. Simul. Mater. Sci. Eng..

[41] Zheng J., Zhang H., Zhou X., Liang J., Sheng L., Peng S. (2014). First-Principles Study of the Structural Stability and Electronic and Elastic Properties of Helium Inα-Zirconium. Adv. Condens. Matter Phys..

[42] Domain C., Legris A. (2005). Ab Initio Atomic-Scale Determination of Point-Defect Structure in Hcp Zirconium. Philos. Mag..

[43] Domain C., Besson R., Legris A. (2002). Atomic-Scale Ab-Initio Study of the Zr-H System: I. Bulk Properties. Acta Mater..

[44] Zhu X., Gao X., Song H., Han G., Lin D.-Y. (2017). Effects of Vacancies on the Mechanical Properties of Zirconium: An Ab Initio Investigation. Mater. Des..

[45] Ford D.C., Zapol P., Cooley L.D. (2015). First-Principles Study of Carbon and Vacancy Structures in Niobium. J. Phys. Chem. C.

[46] Cerdeira M.A., Palacios S.L., González C., Fernández-Pello D., Iglesias R. (2016). Ab Initio Simulations of the Structure, Energetics and Mobility of Radiation-Induced Point Defects in Bcc Nb. J. Nucl. Mater..

